# Diagnostic Challenges in Disseminated Mycobacterium szulgai Infection: A Case Report in the Setting of Advanced HIV Infection

**DOI:** 10.7759/cureus.108067

**Published:** 2026-04-30

**Authors:** Maya Al Salti, Aisha Al Balushi, Fatma Al Farsi, Sultan Al Lawati

**Affiliations:** 1 Medical Laboratory, Rustaq Hospital, Rustaq, OMN; 2 Infectious Diseases, Rustaq Hospital, Rustaq, OMN

**Keywords:** disseminated infection, non tuberculous mycobacteria (ntm), osteo-myelitis, people living with hiv/aids, rifampicin‑based therapy

## Abstract

*Mycobacterium szulgai* is a rare, slow-growing non-tuberculous mycobacterium (NTM), accounting for less than 0.2% of NTM isolates worldwide. It most often presents with pulmonary disease resembling tuberculosis, while disseminated infection is exceptionally uncommon and typically occurs in immunocompromised hosts.

We report a 34-year-old male newly diagnosed with advanced HIV infection who presented with painful cutaneous nodular lesions, intermittent fever, anorexia, and significant weight loss. Initial investigations suggested cutaneous nocardiosis or fungal infection, with cultures yielding *Aspergillus niger* and *Corynebacterium* species. Despite antifungal and antibacterial therapy, his condition progressed to multifocal cutaneous lesions, oral mucosal involvement, and osteomyelitis of the tibia and fibula. An extended microbiological evaluation of a bone biopsy revealed acid-fast bacilli, and cultures identified *M. szulgai* via *rpoB *gene sequencing and matrix-assisted laser desorption/ionization time-of-flight (MALDI-TOF) mass spectrometry (MS). Further imaging and bronchoalveolar lavage confirmed disseminated infection involving lungs, skin, and bone. The patient was treated with rifampicin, ethambutol, and moxifloxacin in combination with antiretroviral therapy. After six months, complete resolution of cutaneous lesions, regression of pulmonary nodules, and normalization of inflammatory markers were achieved. Therapy was planned for 12-18 months.

This case underscores the diagnostic complexity of disseminated *M. szulgai* infection in advanced HIV. Initial misinterpretation as nocardiosis or fungal disease delayed diagnosis, highlighting the importance of extended microbiological workup when conventional therapies fail. Definitive identification through molecular sequencing and MALDI‑TOF MS was crucial. The patient’s favorable response to a rifampicin‑based multidrug regimen emphasizes the need for early recognition and prolonged therapy. This report contributes to the limited literature on disseminated *M. szulgai* and reinforces the importance of considering atypical mycobacteria in immunocompromised patients.

## Introduction

*Mycobacterium szulgai *is a rare, slow‑growing non‑tuberculous mycobacterium (NTM) that has been implicated in pulmonary, cutaneous, and articular infections [1]. Although infrequently encountered in clinical practice, it is clinically significant because its presentation often mimics *Mycobacterium tuberculosis *or other opportunistic pathogens, creating diagnostic challenges, particularly in immunocompromised patients [2]. While pulmonary disease is the most common manifestation, disseminated infection is exceptionally uncommon and typically occurs in severely immunocompromised hosts. This report adds to the limited literature by describing a case of disseminated *M. szulgai *with multi‑organ involvement, including lungs, skin, and bone, in the setting of advanced HIV. By highlighting the overlap with tuberculosis and other opportunistic infections and demonstrating the importance of extended microbiological workup, this case underscores the need for heightened clinical awareness of atypical mycobacteria in vulnerable patient populations.

## Case presentation

**Figure 1:**
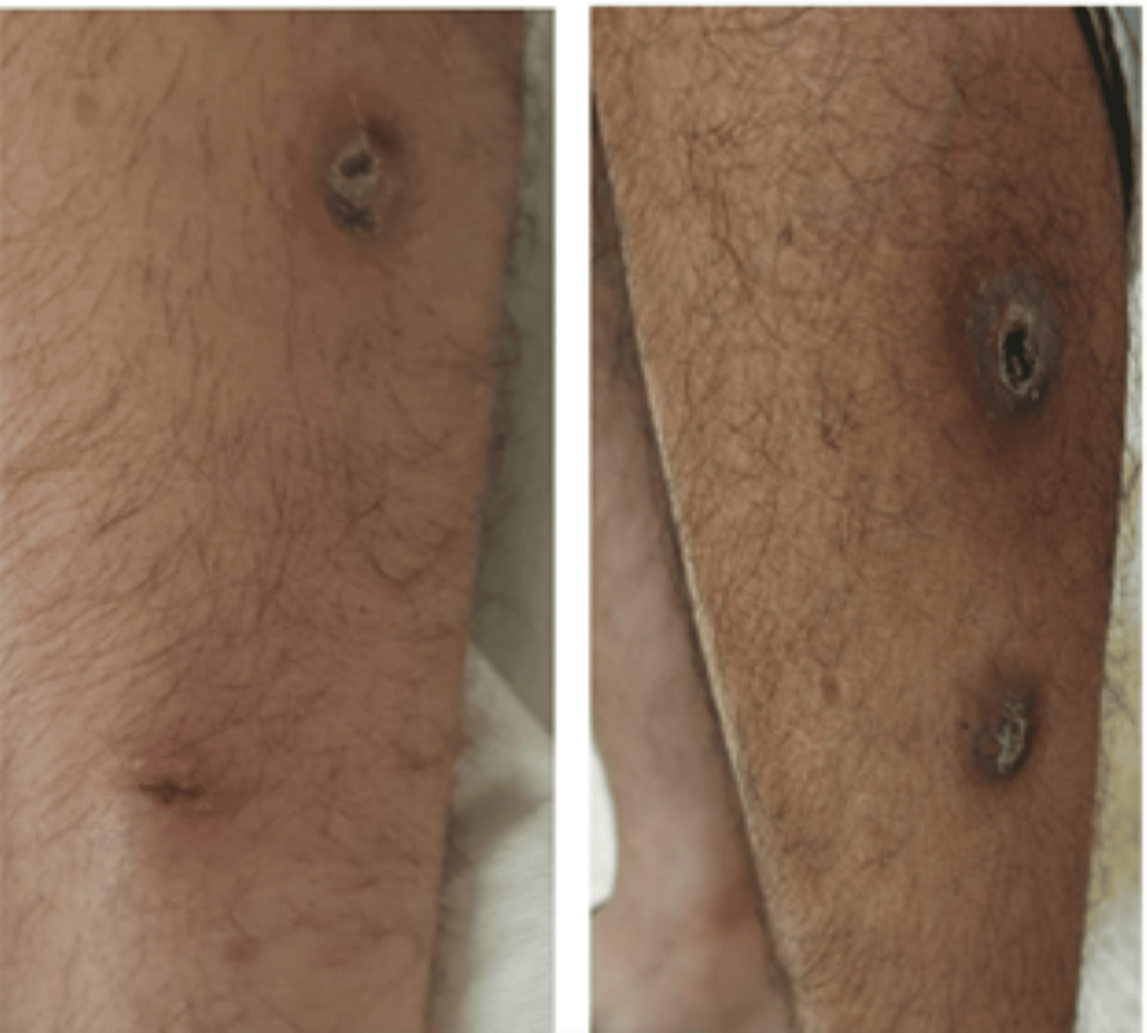
Clinical photograph showing nodular cutaneous lesions with necrotic bases and scant discharge.

A 34‑year‑old male, with no prior medical history, presented with painful cutaneous lesions localized to the lower limbs (Figure 1). The nodular lesions, approximately 1.5 cm in diameter, were distributed along the lateral aspect of the left leg. They exhibited shallow necrotic bases, sloping edges, and scant serous discharge. No regional lymphadenopathy was detected. The patient reported intermittent febrile episodes, anorexia, and significant weight loss. He denied respiratory or gastrointestinal symptoms, recent travel, or substance use. Notably, he had regular contact with domestic cattle.

Initial laboratory investigations revealed neutropenia (0.58 ×10³/µl, normal 2-7.5) and lymphopenia (1.25 ×10³/µl, normal 1.5-4.5), with elevated inflammatory markers (ESR 50 mm/hr, normal 0-15; CRP 110 mg/L, normal <5). Hemoglobin and platelet counts were within normal limits (Table 1). Serological testing demonstrated HIV positivity with a viral load of 191,000 copies/ml, while antinuclear antibody (ANA), antineutrophil cytoplasmic antibodies (ANCA), interferon gamma release assay (IGRA), syphilis, cytomegalovirus (CMV), Epstein-Barr virus (EBV), and *Toxoplasma* serologies were negative (Table 2). 

**Table 1:** Summary of laboratory tests at presentation Hb: hemoglobin, WBC: white blood cells, ESR: erythrocyte sedimentation rate, CRP: C‑reactive protein

Test name	Result	Unit used	Normal range	Interpretation
Hb	12.03	g/dl	11.5-15.5	Normal
WBC	2.5	10x3 /ul	2.2-10	Normal
Neutrophils	0.58	10x3 /ul	2-7.5	Neutropenia
Lymphocytes	1.25	10x3 /ul	1.5-4.5	Lymphopenia
Platelet	2.88	10x9 /l	140-400	Normal
ESR	50	mm/h	0-15	Elevated
CRP	110	mg/l	0-5	Elevated

**Table 2:** Summary of Laboratory test at presentation RH: rhumatoid factor, ANA: antinuclear antibodies, ANCA: antineutrophil cytoplasmic antibodies, HIV: human immunodeficiency virus, HBV: hepatitis B virus, HCV: hepatitis C virus, CMV: cytomegalovirus, EBV: Epstein-Barr virus,  IGRA: interferon-gamma release assay, EIA: enzyme immunoassay

Test name	Result
RH	Negative
ANA	Negative
ANCA	Negative
HCV antibodies	Negative
HBV profile	Immunized
HIV serology	Positive
HIV Viral load	191000 Copies/ml
IGRA test	Negtaive
Syphilis screening EIA	Negative
CMV serology	Negative
EBV serology	Negative
Toxoplasma serology	Negative

**Figure 2:**
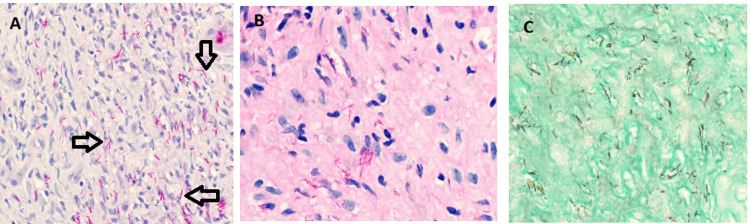
Skin biopsy histopathology stains A: Ziehl-Neelsen (ZN) stain (arrows show acid‑fast bacilli (AFB)), B. Periodic acid-Schiff-diastase (PASD) stain, C. Gomori's methenamine silver stain

Skin biopsy cultures yielded *Aspergillus niger *and *Corynebacterium *species. Histopathology demonstrated mixed acute and chronic inflammation, with fungal stains positive for microorganisms (Figure 2). Differential diagnoses included cutaneous nocardiosis and actinomycotic infection. Empiric therapy with trimethoprim‑sulfamethoxazole and amoxicillin‑clavulanate was initiated. After one week, lesions progressed with new oral mucosal involvement and persistent fever. Inflammatory markers rose further (ESR 109 mm/hr). Therapy was escalated to oral voriconazole for suspected primary cutaneous aspergillosis, alongside antiretroviral therapy (ART) (tenofovir, emtricitabine, dolutegravir).

Two weeks later, partial improvement was noted, but ultrasound revealed multiple subcutaneous collections bilaterally with cortical destruction of the tibia and fibula, suggestive of multifocal osteomyelitis. MRI confirmed bilateral tibial osteomyelitis involving the meta‑diaphysis, associated fibular inflammation, and small abscesses. Bone biopsy demonstrated fibro‑collagenous tissue with necrotic bone, no granulomas, and negative Ziehl-Neelsen (ZN), modified ZN, and Periodic acid-Schiff-diastase (PAS) stains. Acid‑fast bacilli smear was positive, but GeneXpert® (Cepheid, Sunnyvale, USA) excluded *Mycobacterium tuberculosis*. Extended culture identified *Mycobacterium szulgai *via *rpoB *gene sequencing and MALDI‑TOF MS. Susceptibility testing was not performed.

Chest CT revealed bilateral apical reticulonodular opacities, multiple cavitating nodules, a left apical mass, and central bronchiectatic changes. Bronchoscopy with bronchoalveolar lavage (BAL) culture confirmed *M. szulgai*, while cytology excluded malignancy, fungi, *Pneumocystis jirovecii*, and nocardiosis. A final diagnosis of advanced HIV infection with disseminated *M. szulgai *involving skin, bone, and lungs was established.

**Figure 3:**
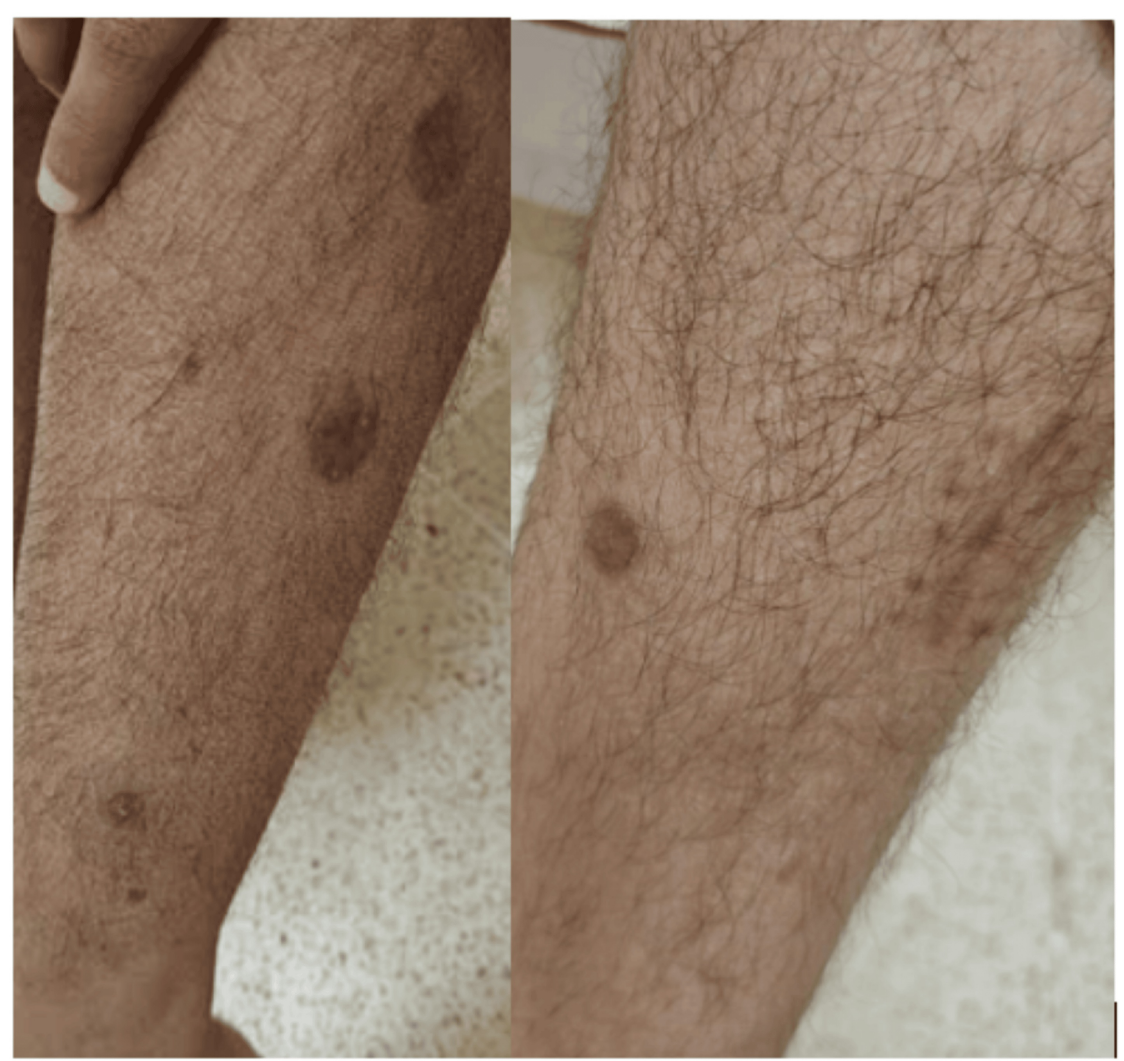
Resolution of the cutaneous lesions after six months of therapy

The patient was commenced on rifampicin, ethambutol, and moxifloxacin, in addition to ART. Within one month, cutaneous lesions improved and inflammatory markers normalized. At six months, complete resolution of skin lesions, regression of pulmonary nodules, and undetectable HIV viral load were documented (Figure 3). Antimycobacterial therapy was planned for a total duration of 12-18 months.

## Discussion

*Mycobacterium szulgai *is a rare, slow‑growing, non‑tuberculous mycobacterium (NTM) first described in 1972. It accounts for less than 0.2% of all NTM isolates worldwide and most commonly presents with pulmonary disease resembling tuberculosis [3]. Extrapulmonary and disseminated infections are exceptionally uncommon, typically occurring in immunocompromised hosts [4-6]. Cutaneous and osteoarticular involvement has been reported rarely, usually in immunocompromised patients or following skin trauma [5]. Clinically, cutaneous disease may be misdiagnosed as cellulitis or other dermatological conditions, presenting with tender erythematous nodules, indurated skin, abscesses, sinus tracts, or ulcerations, sometimes in a sporotrichoid pattern [7]. Environmental reservoirs include snails, aquarium water, swimming pool water, and tropical fish [8].

*Nocardia *infection (nocardiosis) is an important differential diagnosis, with diverse clinical presentations involving the lungs, skin, and brain. Cutaneous nocardiosis may arise from direct inoculation via soil or organic matter, or through hematogenous dissemination in immunocompromised individuals [9]. It can mimic bacterial soft tissue infections, sporotrichosis, or actinomycetoma. Superficial forms resemble cellulitis, lymphocutaneous disease presents as nodules along lymphatic pathways, and actinomycetoma manifests as chronic, destructive lesions with draining sinuses and sulfur granules. Multifocal lesions often indicate dissemination [10]. In this case, the overlapping clinical features complicated the initial diagnosis.

Diagnosis of cutaneous NTM disease is complicated by its heterogeneous presentations, indolent growth, and clinical similarity to other infections such as nocardiosis, fungal disease, or bacterial cellulitis. In immunocompromised patients, particularly those with HIV, progressive lesions unresponsive to conventional therapy should prompt early consideration of NTM. The isolation of *Aspergillus *species from the initial skin biopsy, together with positive fungal stains, created significant diagnostic uncertainty. However, this was ultimately considered contamination rather than true infection, as the organism was recovered from only a single culture plate and was not detected in subsequent bone biopsy or bronchoalveolar lavage samples. The absence of corroborating microbiological or histopathological evidence supported the interpretation of contamination, rather than colonization or coexisting infection. This highlights the importance of correlating culture results with clinical, radiological, and histopathological findings to avoid misdiagnosis and inappropriate therapy.

Histopathologically, *Nocardia *demonstrates thin, branching filaments, while mycobacteria are bacillary. However, heavy burdens of atypical mycobacteria may align to mimic filamentous branching, leading to misinterpretation [11]. 

Microbiologically, mycobacteria are fastidious organisms requiring special media and prolonged incubation. Slow‑growing species such as *M. szulgai *typically appear after 2 weeks, with mature colonies at 3-8 weeks [12]. Identification relies on advanced methods: MALDI‑TOF MS provides high‑confidence identification [13,14], while *rpoB *gene sequencing offers superior discriminatory power [15]. Histopathology usually shows necrotizing or non‑necrotizing granulomatous inflammation, but immunocompromised patients may lack granulomas or visible organisms [16].

Treatment of *Mycobacterium szulgai *generally requires a combination of at least three anti‑tuberculous agents. Because this organism is rare, clinical evidence to guide therapy is limited. Case series suggest that *M. szulgai *is susceptible to most agents except isoniazid [17]. A systematic review of 44 patients reported favorable outcomes in 85% when regimens included rifampicin and a macrolide, with treatment durations of ≥12 months [18]. The role of surgical intervention remains uncertain and is not well established in the management of this infection [19].

In our patient, susceptibility testing was not performed. Therefore, the regimen of rifampicin, ethambutol, and moxifloxacin was selected based on published susceptibility patterns and clinical experience. Rifampicin was included as it is consistently reported to be active against *M. szulgai *and forms the backbone of most successful regimens [18,19]. Ethambutol was chosen for its activity against slow‑growing mycobacteria and its established role in NTM therapy [18]. Moxifloxacin, a fluoroquinolone with documented efficacy in NTM infections, was added to provide a third active agent, particularly in the absence of macrolide susceptibility data [19]. This combination offered broad coverage while avoiding isoniazid, to which resistance is common [18].

The patient responded well to this regimen. After six months of treatment, his cutaneous lesions had completely resolved, and pulmonary nodules showed marked regression. Therapy was planned for a total duration of 12-18 months, consistent with recommendations for prolonged treatment in disseminated NTM infection [19].

## Conclusions

This case illustrates the diagnostic complexity and clinical significance of disseminated *Mycobacterium szulgai *infection in the setting of advanced HIV disease. Although *M. szulgai *is an uncommon non‑tuberculous mycobacterium, it has the potential to cause severe, multi‑organ involvement, including cutaneous, osteoarticular, and pulmonary disease, particularly in immunocompromised hosts. The initial misdirection toward nocardiosis and fungal infection underscores the importance of maintaining a broad differential diagnosis and pursuing extended microbiological workup when conventional therapies fail. Definitive identification through molecular sequencing and MALDI‑TOF MS was crucial in establishing the diagnosis.

While this is a single case, it demonstrates that even delayed recognition of atypical mycobacterial infection in immunocompromised patients can lead to favorable outcomes when appropriate multidrug therapy is initiated. The patient’s response to a rifampicin‑based regimen, combined with antiretroviral therapy, highlights the value of comprehensive evaluation and prolonged treatment in achieving resolution. This report adds to the limited literature on disseminated *M. szulgai *infection and reinforces the need to consider atypical mycobacteria in immunocompromised patients with progressive lesions unresponsive to standard therapy.
